# Sign, Signifier and Signified in Schrödinger's box

**DOI:** 10.1016/j.mex.2024.103047

**Published:** 2024-11-13

**Authors:** Azeena Parveen, Vineeth Radhakrishnan

**Affiliations:** School of Social Sciences and Languages, Vellore Institute of Technology, Vandalur – Kelambakkam Road, Chennai 600127, Tamil Nadu, India

**Keywords:** Quantum social science, Quantum arbitrariness, Signification, Sign-signifier entanglement, Superposition, Signification in quantum perspective

## Abstract

This study of the article proposes an interdisciplinary approach, integrating quantum mechanics and structuralism to elucidate the complex dynamics of signification. By paralleling the ambiguous nature of subatomic particles with the linguistic system, the study examines the arbitrary relationship between sign, signifier, and signified through quantum principles. Key concepts from quantum mechanics, such as wave-particle duality, superposition, entanglement, and observer effect, are applied to Saussure's theory of signs, revealing intriguing analogies between the two domains. The research critically examines the uncertain aspects of lingual systems, using Saussure's theory and Schrödinger's cat experiment to illuminate the complex relationships between signs, signifiers, and signifieds.

This approach can enhance our understanding of language, cognition, and the human experience by shedding light on the intricate dynamics of meaning-making in human communication.

• By applying quantum principles to Saussure's framework of sign, signifier, and signified, the study attempts to understand the intricate mechanisms that govern how meaning is created in human minds.
• Exploring the parallels between linguistic ambiguity and quantum theory reveals the complex interplay between language, thought, and reality, and how they influence one another.
• This approach not only enhances our understanding of quantum social sciences but also offers new perspectives on how language shapes our perceptions, interactions, and understanding of the world around.

## Graphical abstract

**Figure:**
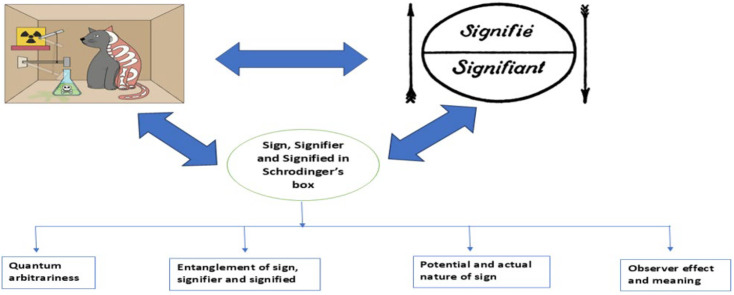

Specifications table

**Table:** 

Subject area:	Psychology
More specific subject area:	*Meaning making process in quantum perspective*
Name of your method:	Signification in quantum perspective
Name and reference of original method:	Saussure's Structuralism
Resource availability:	*None*

## Background

The bridging of multiple disciplines in contemporary research dissolves traditional boundaries between art and science, resulting in new perspectives. By merging Quantum Mechanics and Social Science, scholars like Alexander Wendt and Karen Barad pioneer fresh approaches to understanding complex social phenomena. This interdisciplinary convergence reveals profound connections between the fundamental elements of quantum physics and the structural fabric of social science. The study's approach is grounded in the recognition that the fundamental elements of quantum physics - matter, energy, and information, are inextricably linked to the structural framework of social science [1].

### Quantum mechanics

Quantum theory explores the subatomic realm, yielding groundbreaking studies that expose the intrinsic ambiguity of particles. Research in quantum mechanics reveals the wave-particle nature of subatomic elements, inherently dualistic.

Thomas Young's double slit experiment demonstrated the wave behavior of subatomic elements, challenging classical notions. Physicists like Louis De Broglie, Max Planck, Schrödinger, Heisenberg, and Niels Bohr further explored the ambiguous nature of subatomic particles. This groundbreaking research in modern physics shattered the classical physics' absolute reality paradigm. Quantum theory's focus on probability and potentiality replaced absolute truth, revolutionizing scientific understanding. The inherent ambiguity of quantum mechanics spawned Quantum Social Science, reconciling the complexities of human behavior with the probabilistic nature of reality.

Karen Barad's *Meeting the Universe Halfway* (2007) introduces the concept 'Agential Realism', linking social entanglement to quantum entanglement through ‘intra-action’ [2]. This concept reveals how entities become interconnected and inseparable. Building on this idea, Emmanuel Haven and Andrei Khrennikov's *Quantum Social Science* (2012) applies quantum mechanics' probabilistic nature to economics, psychology, and finance, exploring how uncertainty and entanglement influence human behavior and social systems [3]. Alexander Wendt, in his famous book, *Quantum Mind and Social Science* (2015) studies human consciousness through the quantum phenomenon [4].

## Realm of consciousness

Panpsychism, a philosophical framework, posits that consciousness permeates the universe, encompassing both animate and inanimate entities, thereby attributing a mental state or consciousness to all existence [5]. Alfred North Whitehead, a renowned mathematician and philosopher, contends that reality is contingent upon ‘prehension,’ a concept denoting experiential phenomena [6]. Furthermore, Bertrand Russell's Russellian Monism postulates that consciousness is inherent in all material entities, including microparticles, grounded in phenomenological experience [7]. Notably, panpsychistic notions have been explored since antiquity, with Plato's philosophical treatises foreshadowing modern panpsychism [8].

The philosophical discourse on consciousness has led to the proposition that microparticles possess consciousness. The seminal double-slit experiment conducted by Thomas Young revealed the enigmatic behavior of microparticles, exhibiting both particle-like and wave-like properties contingent upon observational conditions. This phenomenon has spawned the hypothesis that electrons possess consciousness, fuelling the enduring paradox surrounding their seemingly intentional selection of trajectory or slit [9].

In the famous two-slit experiment used to illustrate the wave/particle duality, photons behave quite differently depending on whether, before detection, they are offered the chance to pass through one slit in a screen or through two. If only one slit is open, they behave like particles, hitting the detecting surface like a stream of so many bullets. If two slits are open, they behave like waves, passing through both slits and creating a typical interference pattern on the other side. They seem to ''know" which aspect of their double-sided nature is called for by the experiment and behave accordingly. [10]

David Bohm, a renowned American physicist, presented a metaphysical examination of the correlations between subatomic particles in his seminal work, *Wholeness and the Implicate Order.* Bohm's analysis revealed that spatially separated electrons exhibit an intriguing interconnectedness, wherein their mutual influence persists despite physical distance. Specifically, the motion of one electron instantaneously affects the other, implying a rapid transmission of information. This phenomenon is encapsulated in Bohm's concept of the ‘implicate order,’ which posits that reality is inherently holistic, with ``everything enfolded into everything'' ([11], p. 57). This philosophical framework, aligned with panpsychism and the enigmatic consciousness debate surrounding subatomic particles, lays the groundwork for exploring the signification process through the lens of Schrödinger's thought-provoking experiment.

## Significance of the method

The meaning-making process in linguistics has been a pivotal research focus, with scholars exploring ambiguity and uncertainty through interdisciplinary approaches. Notably, the study ``Signs of Probability: A Semiotic Perspective on the Heisenberg Principle'' [12] examines the constructed reality through the interplay between semiotics and Heisenberg's uncertainty principle. This research highlights how quantum mechanics imbues reality with probabilistic nature, as encapsulated by Heisenberg's principle, with semiotics defining the probabilistic aspects.

Contemporarily, linguistic inquiry is situated within cognitive frameworks. Pragmatics, the contextual study of language, is integrated into scientific cognition by Carston [13], investigating implicit and explicit meaning construction. Furthermore, the study ``Inner Speech and the Linguistic Sign: Toward a Quantum Semiology'' [14] probes the 'sign' and 'speech' through Neuroquantology. This research situates brain duality within thermofield dynamics, illustrating quantum semiology's capacity for sign superposition in communication. This convergence of quantum theory and interdisciplinary research fosters novel philosophical perspectives.

Broekaert's study, ``The Tacit `Quantum' of Meeting the Aesthetic Sign; Contextualize, Entangle, Superpose, Collapse or Decohere'' [15], explores the ambiguous nature of signs (bearing multiple meanings) in texts, images, and art, drawing parallels with quantum mechanics' fundamental aspects: contextuality, entanglement, superposition, collapse, and decoherence. By integrating quantum theory into semiotics, this research reveals the philosophical nexus between cognition and symbolically represented meaning in art and literature.

The study ``Quantum Structure in Cognition: Human Language as a Boson Gas of Entangled Words'' by Aerts and Beltran [16] investigates human language through the lens of quantum mechanics. Utilising Winnie the Pooh as a Boson gas analogy, words and their frequencies are mapped onto energy levels, with cognition represented as ‘quantum human thought.’ This framework analyses cognition and concepts at varying energy levels through quantum mechanical principles.

Research has further evolved to illustrate the entanglement of STEM disciplines with Arts and Humanities. Richard's study, ``Superposition of Quantum Linguistics on Literary Criticism Observing Harold Bloom's Recognition of Noam Chomsky's Literature-As-Genetics: ``When One Speaks a Language, One Knows a Great Deal That Was Never Learned'' [17], demonstrates the superposition of syntactic elements in literary works through quantum linguistics' principles of superposition and entanglement.

In quantum information theory, Heunen et al.'s ``Quantum Physics and Linguistics: A Compositional, Diagrammatic Discourse'' [18] underscores the significance of interdisciplinary research. This study proposes that various disciplines are interconnected by examining category theory in mathematics, which facilitates understanding quantum information theory, quantum protocols, and their relation to computational linguistics, encompassing semantics and grammatical structure.

This research augments the quantum social science landscape by paralleling Saussure's signification paradigm with Schrödinger's seminal thought experiment and the mystifying dynamics of subatomic particles. This innovative approach significantly enriches the existing body of contemporary scholarship in the field.

The key contributions of the study are:

1. Expansion of perspectives in social science, offering a nuanced understanding of human behaviour.
2. Provides academicians to analyse the signification process, gaining insight into how meaning is constructed.
3. Reveals how the mind operates in meaning-making, mirroring the wave-particle duality of quantum mechanics.

## Method details

In human language, meaning is unconsciously negotiated through the complex interplay of signs, signifiers, and signified, governed by tacit mutual agreements. However, these agreements are inherently uncertain, fluid, and susceptible to change as they adapt to the dynamic evolution of people and culture. Consequently, human communication is plagued by inherent ambiguity. The transfer of thoughts or information from one individual to another through any medium is inherently imperfect, as the intended meaning is filtered through the recipient's unique perspective, experiences, and biases. This subjective interpretation process renders the original message vulnerable to misinterpretation.

For instance, consider Person A's statement, ``Sex is life.'' Person B's mind may construe this phrase in a provocative or lascivious manner, whereas Person A's intended meaning might be entirely distinct, perhaps referencing the biological inception of life. This disparity highlights the profound ambiguity inherent in language. So, the transmitted information exists in a state of semantic superposition, remaining ambiguous and open to multiple interpretations until the recipient's mind imposes meaning upon it, effectively collapsing the interpretive possibilities.

This study integrates Quantum theory and Structuralism to explore the human mind's role in meaning-making. Ferdinand De Saussure's Structuralism views language as a system of interconnected signs. He asserts, signs exist in co-relation with each other and do not exist in isolation. A language consists of sign, signifier and signified respectively. Saussure's first aspect is that a sign's meaning is arbitrary, derived from relations and contrasts with other signs, not from its referent [19]. This arbitrary signifier-signified relation parallels quantum mechanics’ principles.

### Sign in quantum superposition

**Fig. 1:**
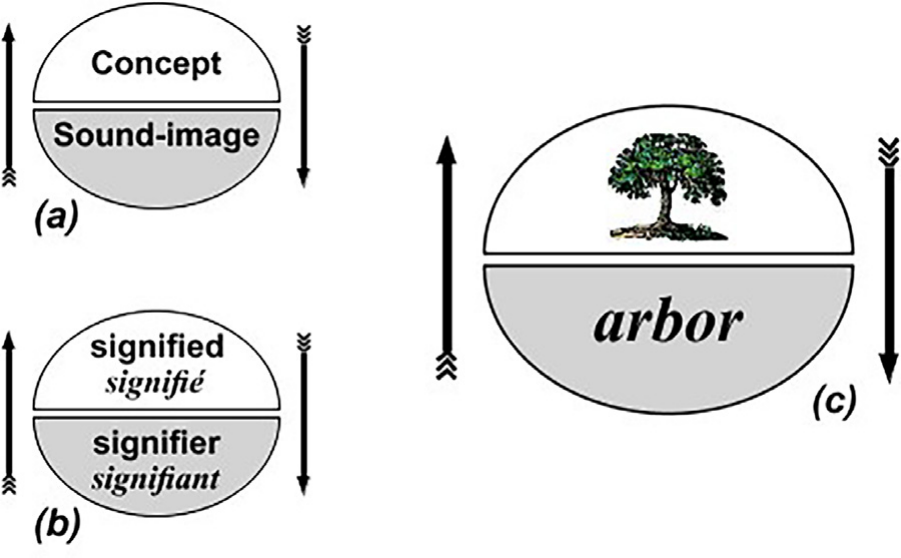
Saussure's signifier and signified [21].

**Fig. 2:**
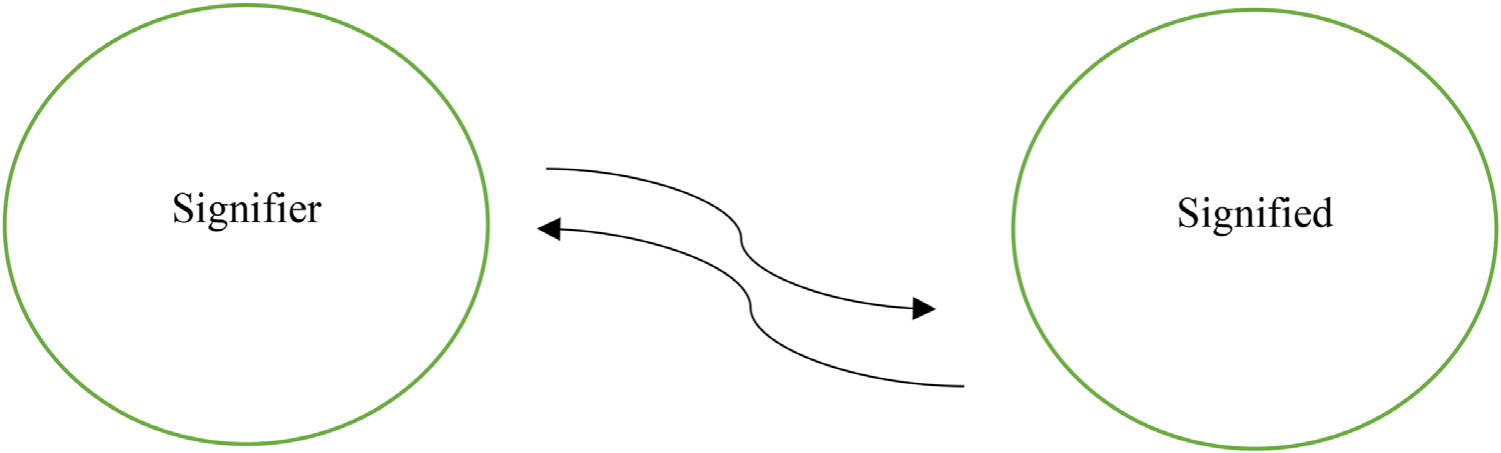
Entanglement of the signifier and signified.

**Fig. 3:**
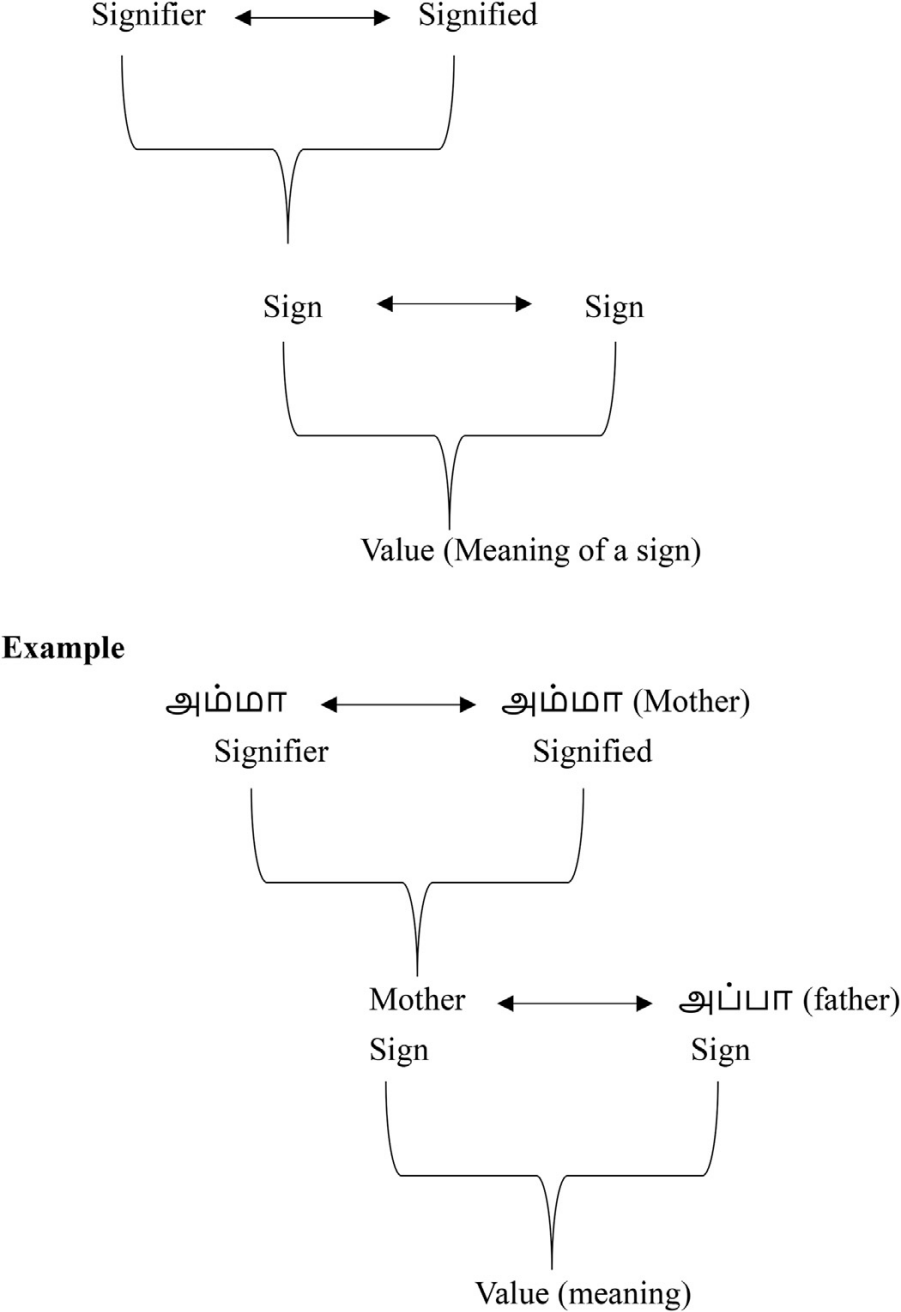
Value of sign through the entanglement process.

**Fig. 4:**
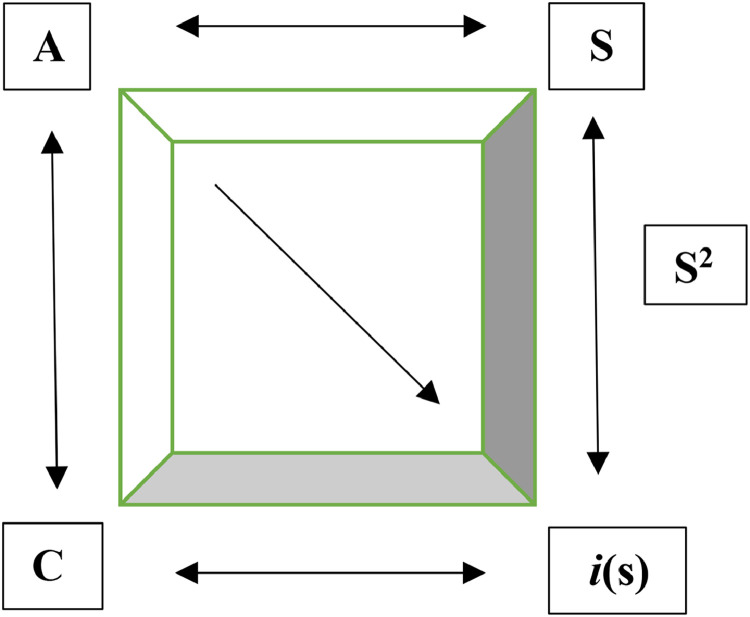
Self within the box.

In quantum mechanics, superposition allows particles to exist in multiple states simultaneously [1]. However, measuring their position collapses this wave-function, sparking debate. Schrödinger argued that wave-function collapse results from particle interactions, not observer intervention [20]. Similarly, Saussure's Structuralism suggests that a sign's meaning emerges from its relationships with other signs, not from external influence. This parallel highlights how meaning arises from internal interactions, whether in quantum mechanics or linguistics (Fig. 1, Fig. 2, Fig. 3, Fig. 4 and Table 1, Table 2, Table 3, Table 4).

### Schrödinger's cat experiment

Erwin Schrödinger's famous thought experiment, ``Schrödinger's Cat,'' illustrates quantum superposition. A cat, radioactive material, poison, and a Geiger counter are placed in a box. If an atom decays, the Geiger counter triggers poison, killing the cat. If not, the cat lives. Since the cat's fate depends on atomic decay, it exists in both alive and dead states simultaneously, mirroring the atom's superposition [22]. This thought experiment challenges Copenhagen's view that observer intervention collapses the wave function. Schrödinger argues that wave function collapse results from internal interactions, not external observation.

### Quantum arbitrariness of sign

Schrödinger's cat and Saussure's Structuralism share an intriguing parallel. Just as the cat's fate is arbitrarily tied to the radioactive atom's decay, the signifier and signified in language are connected by an arbitrary relationship. The cat's life or death isn't inherently linked to the atom, yet its state depends on it. Similarly, the signifier (word) and signified (concept) are not inherently connected, but their meaning is derived from this arbitrary relationship.

Saussure's example of the word ``tree'' illustrates the arbitrary nature of signs. The sound image (signifier) has no inherent connection to the concept (signified), and its meaning isn't derived from physical properties like branches or leaves. Instead, its meaning emerges from contrast with other signs like ``dog'' or ``cat''. This arbitrariness highlights the uncertain, random connection between signifier and signified. Although their relationship is arbitrary, the sign (signifier + signified) relies on other signs to convey meaning, demonstrating a web of interdependent relationships.

### Example

The relationship between the sound image (signifier) and the concept or meaning (signified) is arbitrary. The same signified can be represented by different signifiers across languages.

For example, In Tamil a lion is sounded as  /siŋɡam/ and Le lion /lu ljɔ̃/ in French, and so on.

Although the signifiers differ, all represent the same signified, ``lion''. This reveals the arbitrary nature of language, where the connection between sound and meaning is not inherent.

Different signifiers can represent the same signified, demonstrating the arbitrary relationship between sound and meaning in language. All signifiers are susceptible to change over time.

Eg: thou (old English) – you (modern English)

### Quantum entanglement

Quantum entanglement describes correlated particles that cannot be measured independently, exhibiting connected behavior even at vast distances. Einstein dubbed this as ‘spooky action at a distance’ due to its faster-than-light communication [23]. Schrödinger considered entanglement, “the characteristic trait of quantum mechanics that enforces its entire departure from classical lines of thought” [24]. Applying entanglement's phenomenological aspects to signification reveals an entangled relationship between signifier and signified. Just as entangled particles are inextricably linked, the meaning of a sign emerges from the interconnectedness of its constituent parts, illustrating a non-classical, holistic understanding of language.

The process of signification is a symbiotic entanglement between the signifier (word/image) and signified (concept/meaning), where they are inextricably linked, influencing and informing each other. This entanglement gives rise to meaning, which is not fixed or inherent, but emerges from the dynamic interplay between the two. Just as quantum entanglement transcends spatial boundaries, the signifier and signified transcend their individual identities, becoming an inseparable whole that generates meaning.

The notion of value... shows us that it is a great mistake to consider a sign as nothing more than the combination of a certain sound and a certain concept. To think of a sign as nothing more would be to isolate it from the system to which it belongs. It would be to suppose that a start could be made with individual signs, and a system constructed by putting them together. On the contrary, the system as a united whole is the starting point, from which it becomes possible, by a process of analysis, to identify its constituent elements. [25].

In Saussure's structuralism, a sign's value emerges from its relationships within the language system. For instance, the term ``sister'' gains meaning through contrast with ``brother,'' ``father,'' and ``mother,'' illustrating an entangled process of differentiation [19]. This signification process can be represented as:

$$S\;\left(\frac{S}{s}\right)\times s=v\;\left(t\right)$$

**Table 1:** Process of signification.

S	Signification
$\left(\frac{S}{s}\right)$	$\frac{Signifier}{Signified}$
s	sign
v(t)	valueofasign(time)

The value v (meaning) of a sign, derived through entanglement and signification, is inherently ambiguous and adaptable, influenced by external dynamics like cultural notions and history. As constructed ideologies encounter cultural phenomena, they evolve, and subjective perspectives shift over time. The distinction between signs, facilitated by their entangled relationships, distinguishes one sign from another, thereby generating meaning for the concept.

## Quantum wave function

Erwin Schrödinger's equation, a fundamental concept in quantum mechanics, utilizes the wave function to predict particle behaviour, offering insights into the probabilistic realm of quantum phenomena. The quantum wave function, denoted by ψ (psi), mathematically describes a particle's wave-like behaviour, capturing its probabilistic nature [26]. Quantum wave function is the mathematical description of the wave characteristics of a particle. The value of the wave function of a particle at a given point of space and time is related to the likelihood of the particle's being there at the time [[1], [26]]

iℏ(∂ψ/∂t)=Hψ

**Table 2:** Wave function.

iℏ	imaginaryunitandPlanckconstant
$\frac{\partial \psi }{\partial t}$	partialderivationofwavefunctioninrelationtotime
Hψ	*Hamiltonian operator – total energy of quantum system and wave function* (psi)

Schrödinger's wave function equation, which describes the probabilistic nature of particles, has parallels in the realm of signs. The sign, signifier, and signified can be seen as existing in a state of probabilistic entanglement, where meaning is not fixed but rather a likelihood. This echoes the wave function's description of particle behaviour, where ψ (psi) represents the probability of finding a particle in a given state [26]. Similarly, the sign's meaning is a probability distribution across possible interpretations, highlighting the inherent ambiguity and contextuality of signification.

$$iV\left(\frac{S}{t}\right)=\;sS$$

**Table 3:** Wave nature of sign.

iV	*Denotes the imaginary and probabilistic nature of the value*
$\frac{S}{t}$	*S denotes the signification process in relation to time as it represents how meaning changes according to cultural phenomenon in mean time*
sS	*s denotes the structural relationship between signifier and signified, S denotes the wave function of a sign*

The signification and value of a sign inhabit the realm of quantum potentiality and actuality. Quantum potentiality represents the pre-measurement state, where particles exist as probabilities, not fixed entities. Conversely, quantum actuality emerges when particles assume a fixed state, collapsing the wave function. This parallels the sign's journey from potential meaning to actualized understanding. The observer effect plays a crucial role, analogous to the linguistic act of interpretation, where the observer's perspective actualizes the sign's meaning, transitioning it from a realm of possibilities to a fixed significance.

A sign resides in a state of potentiality, devoid of fixed meaning. For instance, the term ``tree'' exists as a probability distribution of meanings, unfixed and fluid. This wave function of potential meanings collapses during the signification process, actualizing the sign's meaning through interpretation. Just as observing subatomic particles collapses their wave function, the act of interpretation collapses the sign's probabilistic nature, yielding a specific, context-dependent meaning.

## Example

### Socio-cultural factor

Signs, deriving their value from the reciprocal relationship between signifier and signified, transcend linguistic denotation, conveying rich cultural, social, and historical motifs [27]. Situating linguistic discourse within a psychoanalytic framework reveals the profound influence of language on the human mind's structuration. This notion is exemplified by Jacques Lacan, a renowned psychoanalyst, who introduced seminal concepts such as the mirror stage, the Real, the Imaginary, and the Symbolic.

The condition of the subject, S depends on what unfolds in the Other, A. What unfolds there is articulated like a discourse (the unconscious is the Other's discourse [*discours de l'Autre])* … an articulated question – “What am I there?” – about his sex and his contingency in being… is articulated in the Other in the form of elements of a particular discourse. It is because these phenomena are organized in accordance with the figures of this discourse that they have the fixity of symptoms. ([28], p.183)

According to Lacan, the unconscious mind of an individual is repressed by signs imbued with cultural values, which, through linguistic discourse, construct a reality that constrains personal agency. Similar to Schrödinger's cat, humans are trapped within societal power structures, such as cultural norms, perpetuating repression. For instance, fixed gender attributes dictate rigid roles for men and women, exemplifying sociolinguistic discourse. Sociolinguistics examines language's intersection with cultural forms, generating fixed meanings through signifiers and signified [29].

The figure below illustrates the dual nature of the Real Self, analogous to Schrödinger's cat. The Self's identity is contingent upon sociolinguistic codes imposed by the Big Other. The interplay between *C, A, and Subject A* precipitates the Self's struggle with dual identity. The dark shaded area represents the repressed Self, encompassing the imaginary self and the Real Self.

$$\left(iA\frac{\;S\times i\left(s\right)}{t}\right)\times C=\;\surd S$$

**Table 4:** Factors influencing the dual nature of the self.

iA	iA stands for imaginary Other, denoting Lacan's Big Other.
*S*	Denotes the Real Self (Subject)
*is*	Repressed self and the socially constructed imaginary self
t	Denotes the time based on which the meaning of a sign changes and impacts the self.
C	Sociolinguistic code
√S	Represents the wave-particle duality of self, which is stuck between the Real Self and the imaginary self

## Wave-particle duality of the self

For instance, Sexuality is a socially constructed concept, wherein heteronormativity is privileged and homosexuality is stigmatized as a moral transgression. Contrary to its historical recognition as a natural phenomenon, homosexuality has been erroneously thought as choice [30]. Members of the LGBTQ+ community, particularly gay individuals, are frequently subjected to ridicule and feminization of their behavior, leading to profound mental distress [31]. This poignant example illustrates how the authentic Self (Real Self) becomes entrapped within societal constructs.

Despite having the agency to express themselves, individuals often conceal their queer identity to avoid suffering, thereby existing in a state of dual reality. This dichotomy perpetuates the internal conflict between their true self and the socially imposed identity.

## Observer effect, quantum actuality and potentiality

In quantum mechanics, the observer plays a pivotal role, inherently influencing the behaviour of subatomic particles. The act of observation disrupts the wave-like nature of particles, causing them to exhibit particle-like behaviour. This phenomenon, known as wave function collapse, transitions particles from a state of potentiality to actuality. The observer's presence effectively ``pinpoints'' the particles, assigning them a definite position and reality, illustrating the profound impact of observation on the quantum realm.

The observer effect is the phenomenon in which the act of observation alters the behavior of the particles being observed. This effect is due to the wave-like nature of matter, which means that particles can exist in multiple states simultaneously. When an observer measures a particular property of a particle, they are effectively collapsing the wave-function of that particle, causing it to assume a definite state. [32]

The meaning (v) of a sign is subjective, relying on the individual perspectives of speakers, perceivers, and readers. This parallels the observer effect in quantum mechanics, where measurement assigns definite properties to particles. The ``observed, observer, and apparatus'' [1] interact, influencing behavioural change through ``intra-acting agencies'' [1]. Similarly, the sign's meaning emerges from the dynamic interplay between the sign itself, the interpreter, and their contextual apparatus, highlighting the crucial role of individual agency in shaping signification.

Actualizing the meaning of a sign from ambiguity is referred

Observer→observed(signified)→apparatus(signifier−soundimage)=intra−actingagencies

The concept of intra-acting agencies underscores the dynamic meaning-making process, where observers (speakers, perceivers, readers) navigate language's ambiguity to assign definite meaning by producing or establishing linguistic codes. This process collapses the wave function of possible meanings, mirroring the wave-particle duality in quantum mechanics. Language's uncertainty, akin to wave-like behaviour, is actualized into a fixed entity through the observer's engagement, illustrating the intricate interplay between human psyche and linguistic uncertainty.

## Conclusion

Language is a set of meaningful sounds. The signifier in a language is similar to quantum particles which exists in a superposition of states, exhibiting multiple meanings simultaneously. Its interpretation, like wave function collapse, is subject to change upon the observer (people) over time. The act of observation itself influences the signifier's meaning, illustrating the observer effect. Entanglement principles govern the relationships between signifiers, where the meaning of one affect another. Cultural context serves as the quantum field, mediating interactions and shaping interpretations. In fine, language keeps evolving and remains fluid.

## Limitations

None.

## CRediT author statement

**Azeena Parveen:** Methodology, Conceptualization, Writing – original draft preparation, **Vineeth Radhakrishnan:** Writing - Reviewing and editing.

## Declaration of competing interest

The authors declare that they have no known competing financial interests or personal relationships that could have appeared to influence the work reported in this paper.

## Data Availability

No data was used for the research described in the article.
